# The Influence of Form-Focused Instruction on the L2 Chinese Oral Production of Korean Native Speakers

**DOI:** 10.3389/fpsyg.2022.790424

**Published:** 2022-04-11

**Authors:** Mo Chen, Wenya Li

**Affiliations:** ^1^School of Chinese Studies and Cultural Exchange, Renmin University of China, Beijing, China; ^2^Research Institute of International Chinese Language Education, Beijing Language and Culture University, Beijing, China; ^3^Foreign Language Teaching and Research Press, Beijing, China

**Keywords:** L2 Chinese oral production, form-focused instruction, *focus-on-formS*, *focus-on-form*, Korean native speaker

## Abstract

Form-focused instruction (FFI) can help second language (L2) learners notice the forms of language, which is conducive to the acquisition of linguistic forms. Two types of FFIs had been proposed, including *focus-on-formS* (FonFs) and *focus-on-form* (FonF). Previously, studies on FFI in L2 classroom teaching have focused mainly on the influence of two types of FFIs on the L2 acquisition of grammar and vocabulary. The influence of FonFs and FonF on L2 oral production, however, has been addressed less often. The advantages and disadvantages of different teaching methods in FonFs and FonF have not been well investigated. On the basis of Schmidt’s noticing hypothesis, VanPatten’s input processing hypothesis, and Long’s interactive hypothesis, we investigated the effects of teaching goals, teaching steps, and interactive activities in FonFs and FonF on the complexity, accuracy, and fluency of 32 native Korean speakers’ L2 Chinese oral production. We found that FFI significantly improved the performance of L2 Chinese oral production, although different FFIs had different effects on complexity, accuracy, and fluency. FonF and FonFs could improve both complexity and accuracy, whereas FonF also significantly improved fluency, which was not observed in FonFs. Furthermore, we found that the level of L2 proficiency could modulate the interaction between instruction methods and learning outcomes. For low-proficiency learners, FonF was more helpful for the improvement of fluency. For high-proficiency learners, FonFs was more helpful for the improvement of accuracy. These results demonstrated that teachers should pay attention to the interaction between specific teaching conditions with different L2 proficiency and learning outcomes when implementing FFI. The findings of this study have important implications for the design of procedures and interactive activities of L2 spoken Chinese teaching.

## Introduction

Determining how to integrate linguistic forms into a communicative language teaching (CLT) classroom has always been a great challenge in the field of second language (L2) acquisition. Ellis (1997) categorized the current L2 pedagogical approaches to the teaching of form and meaning of language into two types: meaning-focused instruction (MFI) and form-focused instruction (FFI). The former requires learners to focus on the meaning of expression, whereas the latter includes not only the traditional teaching of linguistic forms but also the teaching strategy of using forms in meaning-based communication activities. Some studies (DeKeyser, 1998; Norris and Ortega, 2000) have reported that MFI does not improve grammar ability, whereas FFI helps learners pay more attention to linguistic forms when they appear in meaningful communicative activities, which is more conducive to language acquisition (Ellis, 2016; Sippel, 2021). In recent years, the research on FFI has shifted from focusing on the impact of FFI on L2 acquisition to the effects of teaching conditions of different types of FFI on learning outcomes (Khezrlou, 2021; Michaud, 2021; Saeed and Reinders, 2021).

The FFI refers to all of the planned or incidental teaching activities that guide learners to pay attention to linguistic forms, including traditional teaching ways based on structural syllabus and CLT (Ellis, 2016). FFI helps learners to acquire language by emphasizing the formal elements of language. Long (1988; 1991; 1996) first proposed two types of formal pedagogical approaches to grammar: one focused on forms (FonFs) and another focused on form (FonF). FonFs refers to the discrete and explicit teaching of syntactic forms based on a structural syllabus (Long, 1988, 2018). FonF, however, refers to a temporary shift in learners’ attention from linguistic meaning to linguistic form triggered by communicative need in the context of meaning-focused communication (Long, 1991). The advantages and disadvantages of these two approaches have raised significant concern and discussion (Ellis, 2016). Some researchers (Doughty and Williams, 1998; Ellis et al., 2002) have proposed that FonFs is a teaching method that does not focus on communication and cannot effectively promote learning, whereas other researchers (e.g., Sheen, 2005) have claimed that FonFs is better than FonF in L2 acquisition. Therefore, an in-depth investigation and discussion of the inconsistency of these studies is necessary.

Note that there are research gaps in previous studies. First, previous studies on FFI have focused mostly on L2 learners in Indo-European languages, such as English, French, German, and Spanish. Few studies have been reported in L2 Chinese learners (Gong et al., 2018, 2020a,b). Second, most of the previous studies on FFI had focused on the specific linguistic items (Afshar, 2021) and have ignored L2 oral competence. For L2 Chinese teaching, the acquisition of oral competence has always been the focus of classroom teaching (Ma et al., 2017; Gong et al., 2021). Much effort is needed for teachers to find suitable strategies to prepare L2 spoken Chinese lessons. It is unclear that the influence of FFI on L2 spoken Chinese. Third, previous studies have paid little attention to the modulation effects of L2 proficiency on learning outcomes based on different pedagogical approaches (Sok et al., 2019). To fill the research gaps and provide guidance for L2 spoken Chinese teaching, we examined the effects of two different types of FFI on the oral production of L2 Chinese learners with different levels of L2 proficiency. We further explored what conditions are most effective for improving L2 Chinese oral competence.

## Literature Review

### Focus on Forms and Focus on Form

In FonFs, the teacher provides opportunities for learners to use syntactic forms to promote the automation of language skills and the accuracy of the linguistic forms (DeKeyser, 1998; Nielson and DeKeyser, 2019). FonFs pays more attention to form and less attention to meaning and is similar to mechanical practice. In FonF, learners’ attention is focused on linguistic meaning, and only when communication difficulties arise do learners shift their attention to form. Learners thus pay attention to the form and meaning of language alternately, which helps them map form to function (Long, 1991; Long and Crookes, 1992).

Ellis (2016) divided FonF into preemptive FonF and reactive FonF, according to whether the focus on form is preemptive or reactive in meaning-focused communicative activities. In preemptive FonF, even if there are no errors in linguistic forms or difficulties in understanding, the teacher or the learner still takes time from communicative activities to focus on the linguistic forms that may potentially cause problems. This time out means that the learner temporarily switches their role from that of language user to that of language learner. In reactive FonF, the teacher gives feedback on learners’ errors in communicative activities so that they learn to use linguistic forms correctly. As a benefit of this mechanism, the teacher and learners incidentally pay attention to form, so their attention is broadened (Ellis, 2001). When communication problems occur, the teacher and learners conduct “negotiation of meaning” or “negotiation of form.” The teacher gives two types of feedback: implicit and explicit. Implicit feedback includes requests for confirmation, clarification, and recasting, whereas explicit feedback includes elicitation, metalinguistic explanation, and explicit correction (Ellis et al., 2002).

In short, FonFs is based on a synthetic syllabus and adopts an explicit way to teach. Language acquisition is regarded as a learning process based on accumulating isolated grammar items. In FonFs, learners integrate the isolated items needed for communication (Long and Robinson, 1998). FonF, meanwhile, presents grammatical knowledge in a communicative context, which is not a simple regression to FonFs. The essential difference between FonFs and FonF is that FonFs separates linguistic elements from the communicative context and endows learners with the role of language learner. In contrast, FonF always takes communication as a prerequisite for learning and regards learners as language users. Therefore, the focus of FonFs is solely on linguistic elements and ensuring that learners gradually improve accuracy, whereas FonF highlights the importance of form–function mapping, which is more helpful in improving fluency and accuracy of speech production (Doughty and Williams, 1998).

### Theoretical Framework

Our study investigated the effects of FonFs and FonF on L2 Chinese oral production with the guidance of the noticing hypothesis (NH), the input processing hypothesis (IPH), and the interaction hypothesis (IH). The NH emphasizes the attention to the linguistic forms. The IPH underscores the comprehension of the meaning attached to the linguistic forms. The IH highlights the meaning and form negotiation in interaction. The assumptions of the three hypotheses and their relationships with our study are discussed next.

#### Noticing Hypothesis

According to the NH (Schmidt, 1990), the premise of language acquisition is that learners must consciously pay attention to the linguistic form in input. Only when learners pay attention to the features of the target language can they store language features into working memory and convert them to “intake” (Schmidt, 1994). Learners then test the hypothesis, rule reconstruction, modification, and output, and finally transform the input into acquired knowledge (Doughty and Williams, 1998; Skehan, 1998). The teaching purpose of FonFs is to make learners notice these linguistic forms. FonF, however, enables learners to notice not only the linguistic forms but also the linguistic meaning. In theory, a linguistic feature is more likely to be internalized only when it receives attention from the learner.

#### Input Processing Hypothesis

The IPH proposes that learners must pay attention to the linguistic forms in language input before they can establish a connection between the form and the meaning of language because of limited attention resources. In theory, learners need not only to notice the linguistic forms but also to understand the meaning underlying those forms (VanPatten, 2007). For FonFs, which focuses on the linguistic forms, learners may not be able to comprehend the meaning of the language features. In contrast, for FonF, the meaning-based communicative activities, learners can process the language features only when they notice the form elements of language features and understand the meaning of those features.

#### Interaction Hypothesis

The IH (Long, 1996) proposes that learners notice the linguistic forms when they are difficult to understand and have a chance to negotiate the linguistic meaning during meaning-based communication. This negotiation helps the learner to highlight the linguistic forms that are hard to understand, notice the gap between the input and their own interlanguage (Schmidt and Frota, 1986), and gives learners the opportunity to produce output. This kind of meaning negotiation focused on specific forms will improve L2 acquisition. For FonFs, the learners have few opportunities for meaning negotiation when they encounter comprehension difficulties. Moreover, when learners have difficulty in the production of the linguistic forms, they may not be able to construct the connection between form and meaning for the lack of negotiation of form. Unlike FonFs, in the meaning-centered communication (i.e., FonF), learners can build connections between form and meaning through the negotiation.

### The Influence of Form-Focused Instruction on L2 Acquisition

Regarding the impact of FFI on L2 acquisition, previous studies focused more on grammar and vocabulary than on L2 oral competence. We examined the progress made in previous studies and analyzed their limitations.

#### The Influence of Form-Focused Instruction on L2 Grammar Acquisition

Previous studies on the impact of FFI on L2 acquisition focused primarily on syntax, covering learners’ native language in English, German, French, and Spanish. Most of these studies found that FFI can promote L2 syntactic acquisition (Koster and Cadierno, 2019; Shabani and Hosseinzadeh, 2019; Trahey and Spada, 2020). The researchers also investigated the teaching conditions that could potentially improve the role of FFI. The comparative study of explicit and implicit teaching has shown that explicit teaching was more beneficial to improve the accuracy of L2 syntactic structure and strengthen syntactic awareness than implicit teaching (Shintani, 2015; Dhiorbhain and Duibhir, 2017). The comparative study of different testing times has shown that compared with post-task and online-task FFI, pre-task FFI was the most effective strategy in improving the complexity, accuracy, and fluency of English subjunctive expression (Michaud, 2021). Khezrlou (2021) found that pre-task FFI and post-task FFI were effective in improving the fluency of English passive expression, while pre-task FFI combined with online-task FFI demonstrated the best performance in improving the accuracy of passive structure.

#### The Influence of Form-Focused Instruction on L2 Vocabulary Acquisition

Previous studies on the impact of FFI on L2 vocabulary acquisition have focused mostly on the differences of the effects of FonFs and FonF on the acquisition of verbs, nouns, plural -*s*, and copula-*be* for L2 English learners. Compared with FonFs, FonF was reported to be more conducive to L2 vocabulary acquisition (Marefat and Hassanzadeh, 2016), and helped learners pay attention to the vocabulary forms (Fuente and Maria, 2006). For example, FonF based on task-based instruction was more effective than FonFs based on present, practice, and production (3P instruction) in the acquisition of L2 English adjectives and plural-*s* (Shintani, 2013, 2015). Contrary to these previous findings, Laufer (2005, 2006) claimed that FonFs was more conducive to L2 vocabulary acquisition because it could make learners pay attention to the correct linguistic forms. Hong and Wang (2016) found that FonFs played a more active role in L2 Chinese lexical collocation and word meaning association than FonF. Interestingly, some studies have found that both FonF and FonFs were effective in L2 vocabulary acquisition (Shintani, 2013; Khezrlou, 2021). Other studies, however, have shown that FonF and FonFs do not have significant effects on some vocabulary items, such as the copula-*be* (Shintani, 2015). Overall, the inconsistency of these results may be explained by the attributes of the target structure selected by the researchers (Xu and Lyster, 2014) and the processing characteristics of different teaching methods (Shintani, 2013).

#### The Influence of Form-Focused Instruction on L2 Oral Acquisition

Existing studies on the impact of FFI on L2 oral acquisition have focused mostly on accuracy and fluency, whereas few have examined complexity. Moreover, the participants of these studies mainly have been L2 English learners. Most studies have found that FFI has a positive impact on L2 oral acquisition (Snellings et al., 2002; Toni and Hassaskhah, 2018). Research on children’s L2 acquisition has shown that FFI is beneficial to improve fluency (Pena and Pladevall-Ballester, 2020) and accuracy (Hyun, 2021). Compared with non-FFI instruction, FFI was more beneficial for college students’ L2 oral grammar, vocabulary, and fluency (Lee, 2016). Form-focused FFI is less effective than meaning-focused instruction in fluency (Arslanyilmaz, 2013). Other studies have focused on the impact of teaching conditions in FFI on L2 oral acquisition. For example, a study on the impact of interactive tasks on L2 oral English acquisition found that single-person tasks were beneficial only to lexical complexity, whereas double-person interactive tasks were beneficial to accuracy, syntactic complexity, and lexical diversity (Li, 2015). Another study on teaching timing found that FonF in the post-task stage improves accuracy, syntactic complexity, and lexical complexity (Li, 2013). A study on the impact of interaction and feedback on L2 oral English revealed that peer interaction and corrective feedback contributed to the improvement of accuracy, but peer interaction was more effective in improving fluency (Sato and Lyster, 2012).

### The Modulation Effects of L2 Proficiency on the Effects of Form-Focused Instruction

L2 proficiency can significantly modulate the effects of FFI. A meta-analysis of the effects of FFI in L2 teaching in the past 35 years found that FFI was beneficial to learners at all L2 levels (Kang et al., 2019). Moreover, the impact of FFI on beginning learners was greater than that of intermediate learners and advanced learners (Kang et al., 2019). The researchers attributed these results to the fact that beginners need more external support for their lack of L2 knowledge. Other studies (Williams, 2001; Nassaji, 2010), however, have found that high-proficiency learners benefit more from FFI and pay more attention to forms than low-proficiency learners. These findings indicated that low-proficiency learners may inhibit attention to forms when they encounter difficulties in decoding and encoding meaning (see VanPatten, 1990). The inconsistency of these conclusions may be related to the explicitness of target language features and the characteristics of outcome measures. In general, although L2 proficiency modulates learners’ participation and the benefits of FFI to some extent (Ellis, 2016), the variable of L2 proficiency is usually underestimated. Most FFI studies have focused on elementary and intermediate learners and rarely have considered advanced learners (Kang et al., 2019; Sok et al., 2019). Moreover, many studies have lacked the standardized measurements of L2 proficiency or have not reported L2 proficiency (Kang et al., 2019; Sok et al., 2019). In summary, the modulation effect of L2 proficiency on the teaching effect of different types of FFI must be explored.

### The Modulation Effects of Outcome Measures on the Effects of Form-Focused Instruction

To a certain extent, outcome measures modulate the effectiveness of the instruction. First, the instruction effects of different FFIs are different for different types of measured knowledge. Early studies mainly have used controlled measures of explicit knowledge (e.g., multiple choice and cloze tests), which helped produce more significant explicit instruction effects. Recent studies have relied more heavily on free production (Kang et al., 2019), which can stimulate learners’ spontaneous use of implicit knowledge and lead to more significant effects of implicit instruction (Spada and Tomita, 2010). Note that the test timing is associated with different degrees of instruction effectiveness. Goo et al. (2015) and Kang et al. (2019) found that both explicit and implicit instruction produced significant immediate learning effects, but the learning effects of implicit instruction lasted for longer than explicit instruction (Goo et al., 2015; Kang et al., 2019). Second, the instruction effects of different types of FFIs also changed with the variable modes of outcome measures. Learners’ performance in the oral mode was significantly better than in the written mode. Most oral measures used selected responses (e.g., multiple choice), whereas most written measures used metalanguage judgment tasks, which may be more difficult than selected responses (Norris and Ortega, 2000). Overall, it seems that if more measures are used, the opportunity is better to observe the instruction effects (Goo et al., 2015). Thus, the outcome measures should reflect not only implicit knowledge but also explicit knowledge. In addition, oral mode measures also have been used in recent studies (Kang et al., 2019). Therefore, these measures should be able to make a comprehensive assessment of oral production to explore the instruction effects of different types of FFIs with different types of measured knowledge.

## The Research Questions

The objectives of this study were to explore the specific conditions for optimizing L2 spoken Chinese teaching, to clarify the modulation effects of L2 proficiency on the learning outcome in different types of FFI, and to identify the pedagogical approaches suitable for high- and low-proficiency learners. In summary, our study examined the following two questions:

RQ1. Do different FFIs (FonFs and FonF) have different effects on L2 Chinese learners’ oral production?

RQ2. Does L2 proficiency modulate the learning outcomes in different pedagogical approaches?

## Research Method

### Experiment Design

This experiment was a three-way mixed analysis of variance (ANOVA), using a within-subjects design with the variable of testing time, including pretest, immediate posttest, and delayed posttest. We also designed two between-subjects variables in this study: the instruction group (FonFs and FonF) and L2 proficiency (low and high). The dependent variables were the oral production measures of complexity, accuracy, and fluency (see Section “Measurement of Dependent Variables” for details).

### Participants

The participants included 32 native Korean speakers who were L2 Chinese learners from Seoul, Busan, Gyeonggi do, and Daegu in South Korea. The participants were between 19 and 30 years old (*M* = 24.06; *SD* = 2.44). Korean native speakers face various challenges when communicating in L2 Chinese, including weak communication initiative, strong dependence on written language, low fluency, and complexity of oral production (Wang and Wang, 2014). In this study, we investigated which teaching conditions in FFI could improve native Korean speakers’ oral production.

The participants were undergraduate or graduate students from Beijing Language and Culture University, Beijing University of Science and Technology, and Capital Normal University. Among the participants, there were 16 males, with an average age of 24.18 years (*SD* = 2.50) and 16 females, with an average age of 24 (*SD* = 2.48). Because Hanyu Shuiping Kaoshi (HSK) is a standardized international Chinese proficiency test for non-native speakers (Zhang, 2018, 2021; Zhang et al., 2020), this study placed learners into either a high-proficiency group or a low-proficiency group based on their HSK grades. Sixteen high-proficiency learners had achieved HSK-5 (*n* = 8) or HSK-6 (*n* = 8) and had studied Chinese for about 3 or 4 years. Sixteen low-proficiency learners had achieved HSK-3 (*n* = 8) or HSK-4 (*n* = 8) and had studied Chinese for about 1 or 2 years.

### Instruction Grouping

All 32 participants were randomly divided into four groups with eight people in each group (see Table 1).

**TABLE 1:** Biographical and proficiency characteristics of the L2 Chinese learners.

Instruction group (4)	L2 proficiency	Number	Sex (female/male)	Age (mean/*SD*)	Learning experiences (mean/*SD*)
FonF	Low	8	6/2	24.1 (3.2)	1.5 (0.5)
	High	8	5/3	25.4 (3.5)	3.5 (0.5)
FonFs	Low	8	6/2	25.3 (3.8)	1.5 (0.5)
	High	8	5/3	25.9 (2.9)	3.4 (0.5)

We used four oral production tasks as pretests (see Section “Instruments” for details). The independent sample *t*-test results (see Table 2) of the pretest did not find any significant difference between the two low-proficiency and two high-proficiency groups in terms of fluency (*t* = −0.632, *df* = 14, *p* = 0.537; *t* = −0.007, *df* = 14, *p* = 0.994), lexical complexity (*t* = 0.160, *df* = 14, *p* = 0.876; *t* = 1.121, *df* = 14, *p* = 0.281), syntactic complexity (*t* = 0.052, *df* = 14, *p* = 0.959; *t* = 0.289, *df* = 14, *p* = 0.777), lexical accuracy (*t* = −0.417, *df* = 14, *p* = 0.683; *t* = 0.275, *df* = 14, *p* = 0.788), and syntactic accuracy (*t* = −0.203, *df* = 14, *p* = 0.842; *t* = 0.275, *df* = 14, *p* = 0.788).

**TABLE 2:** Descriptive statistical results for the pretest.

Proficiency	Instruction group	Fluency	Lexical complexity	Syntactic complexity	Lexical accuracy	Syntactic accuracy	Number
Low	FonFs	4.007 (0.668)	15.924 (3.251)	1.736 (0.232)	0.294 (0.167)	0.323 (0.168)	8
	FonF	4.186 (0.445)	15.690 (2.592)	1.729 (0.264)	0.326 (0.142)	0.337 (0.071)	8
High	FonFs	6.135 (1.227)	24.821 (3.176)	1.962 (0.188)	0.233 (0.064)	0.289 (0.055)	8
	FonF	6.139 (0.476)	23.352 (2.361)	1.928 (0.256)	0.220 (0.118)	0.278 (0.095)	8

### Teaching Procedure

All of the teaching activities in this experiment were undertaken by the same teacher to avoid the bias of different teaching styles on the experimental results. The participants were asked to come together to form temporary groups. The teaching process lasted 2 weeks and consisted of five classes, wherein the last was a review class. Two teaching sessions were conducted in the first week and three were conducted in the second week. Each class lasted 40 min. The differences between FonFs and FonF in teaching procedures in terms of goals, contents, steps, and interactive methods are described in the following sections.

#### Teaching Goals

The teaching goal of FonFs is to enable learners to accurately master the linguistic forms and achieve the processing automation of lexical and syntactic structure, and thus improve the accuracy and complexity of linguistic forms. The teaching goal of FonF is to enable learners to realize the conversion of attention according to communicative needs, and at the same time. to notice fluency, complexity, and accuracy.

#### Learning Content

The learning material consisted of four articles. Each article contained about 225 Chinese characters, 10 new words, and two grammar points that learners had not learned. The topics of the article involved travel planning, online life, and shopping. Learners with the same language level received the same learning materials. To design the learning materials, we considered that learning materials should conform to learners’ existing language level, which not only replicated the ecological validity of a real classroom environment but also conformed to the ethics of teaching experiments. The oral tasks implemented in this study were the same for all learners. Therefore, we strictly controlled the difficulty level of vocabulary and syntactic structure of the learning materials for different language levels with reference to the *Outline of Chinese Vocabulary and Chinese Character Difficulty Level* published by The National Chinese Language Examination Committee (2001) and the *Outline of the Standard and Grammar Level of the Chinese Proficiency* published by The Office of the National Leading Group for Foreign Language Teaching (1996). For example, when learning how to make a travel plan (see Supplementary Appendix 1), the learning materials were designed to be different only in the HSK level of the target words and grammar points, while other contents remained the same. The words and grammar points learned by low-proficiency learners were required by HSK 3–4 levels, and those learned by high-proficiency learners were required by HSK 5–6 levels. Moreover, the oral production tasks in this study did not measure the difficulty level of vocabulary and syntax structures produced by participants in tests, which offset the potential impact of the difficulty level of vocabulary and grammar points in the learning materials to a certain extent. Each participant studied one article in each class. Teaching topics were related to the testing topics, but the tasks were different. For example, the travel planning lesson in the teaching sessions was designed for the student and their friends to go to cities in southern China, whereas the travel planning lesson in the testing phase was designed for their parents’ weekend trip to Beijing.

#### Teaching Steps

The teaching steps for FonFs were introduced as follows: In FonFs, the teacher followed the 3P instruction sequential guideline (Ur, 1996; Ellis, 2001) to teach vocabulary and syntactic rules.

##### Teaching Steps of FonFs

At the presentation stage, the teacher used a picture display and dialog to enable learners to learn new words and syntactic rules by the deduction or induction method. For example, the teacher taught the word “queue” (*排队*) through the dialog method (see the example for word instruction), and used the induction method to teach “from + place A + to + place B” (*从+地方A+去+地方B*; see the example for grammar instruction).

Example of word instruction:

T (Teacher): There are a lot of people when we go shopping. What should we do in this situation?

T: *我们买东西的时候，人很多，这个时候我们要怎么做？*

S (Student): Wait in line.

S: *排队买。*

T: Good! So, we say, “line up to buy something” and “line up to buy tickets.”

T: *很好！所以我们说“排队买东西”、“排队买门票”。*

Example of grammar instruction:

T: Where did they set out from?

T: *他们是从哪里出发的？*

S: Beijing Capital International Airport.

S: *北京首都国际机场。*

T: Where did they go?

S: *去了哪里？*

S: Hangzhou.

S: *杭州。*

T: Please say the complete sentence.

T: *请说完整的句子。*

T and S: They left for Hangzhou from Beijing Capital International Airport.

T and S: *他们从北京首都国际机场出发，去了杭州。*

T: And then? Where have they been?

T: *然后呢？又去了哪里？*

S: Chengdu and Dali.

S: *成都和大理。*

T: Where did they leave for Chengdu and Dali this time?

T: *这次他们是从哪里出发，去的成都和大理呢？*

S: Hangzhou.

S: *杭州。*

T: Please say the complete sentence.

T: *请说完整的句子。*

T and S: This time they set out from Hangzhou to Chengdu and Dali.

T and S: *这次他们从杭州出发，去了成都和大理。*

T (Summary): What’s the common structure between the two complete sentences above?

T (Summary): *我们刚才说的两个完整的句子，在结构上有什么共同点？*

S: They are all “Set out from + place A, go to + place B.”

S: *都是“从哪里出发，去了哪里”。*

T: Very good! This structure is “Set out from + place A, go to + place B” (At this time, the teacher wrote the structure on the blackboard).

T: *很好，所以我们说“从+地方A+出发, 去+地方B”。*

At this stage of practice, the teacher used mechanical drills, such as word filling and sentence replacement, to help learners consolidate their knowledge of vocabulary and grammar. Example drills are as follows:

Please combine the following two sentences into one sentence.

David set off from Shanghai yesterday. David went to Wuhan yesterday.

*大卫昨天从上海出发。大卫昨天去了武汉。*

Answer: David left Shanghai for Wuhan yesterday.

Answer: *大卫昨天从上海出发去了武汉。*

At this stage of development, learners paid more attention to the linguistic forms by completing exercises, such as word selection and retelling.

##### Teaching Steps of FonF

FonF is a crucial feature of task-based language teaching (Ellis, 2016). According to a previous study (Williams, 1995), we defined the three teaching stages of FonF as follows: pre-task stage, task processing stage, and language-focused stage.

In the pre-task stage, the teacher assigned information gap tasks to learners, such as decision-making or reasoning tasks. The teacher provided learners with target language input by introducing requirements and operation methods as well as task purposes. Learners could obtain for L2 input and output from the learning materials. For example, when learning how to make a travel plan, the teacher provided learners with an opportunity for meaning negotiation through task requirements (see the pre-task example).

Pre-task example: How do you make a travel plan?

Two people work together to complete the task and make a travel plan according to the following pictures (select up to three scenic spots), keywords, and content clues.

(I) Pictures of scenic spots (omitted).

(II) Keywords (omitted).

(III) Content clues (see Table 3).

**TABLE 3:** Content clues for a travel plan.

Travel plan
Departure time
Vehicle
Tourist routes
Accommodation arrangement
Payment
Other activities

In the task-processing stage, learners were required to complete tasks and prepare and submit oral reports. Meaning negotiation in group discussion could enable learners to obtain comprehension input. This input would help learners notice the gap between their native language and the target language. Therefore, learners could more deeply process the linguistic forms. The teacher used various interactive ways to provide suggestions and requirements for the learners’ oral report (see Section “Interactive Method”). The teacher did not interfere with the learners’ behavior during the task-processing stage, which would have helped the learners pay more attention to the expression of meaning. Thus, the learners had to pay attention not only to the meaning of language but also to the choice of linguistic forms in the task-processing stage.

In the language-focused stage, the learners had to analyze the linguistic forms in the standard text provided by the teacher. Then, learners were required to use these linguistic forms for the new tasks. The lexical and syntactic structures in the standard texts were underlined or marked in red to strengthen learners’ attention to the linguistic forms.

#### Interactive Method

The interaction between the teacher and learners affected the attention learners gave to linguistic forms (Ellis, 2016). Therefore, the current study controlled the interaction in the different instruction groups. The FonFs and FonF interactive methods are described in greater detail in the following sections.

##### Interaction in FonFs

The FonFs followed an interactive strategy known as initiate-response-feedback (IRF) (Long, 2018). The first step was to initiate, which meant the teacher helped the learners to focus on the linguistic forms through closed questions when presenting the linguistic forms. The second step, response, meant that learners responded to the teacher’s questions. Feedback refers to the teacher’s correction of the answers from the learners. The teacher provided feedback in the form of “yes” or “no” and “right” or “wrong,” and the purpose of this feedback was to solve common problems in learners’ speech expression. In FonFs, the interaction occurred primarily between the teacher and learners, and the topic of interaction was predetermined. Following are some interactive examples of FonFs:

T: Where did David go? (Initiate)

T: *大卫去哪里旅行了？* (Initiate)

S1: He went to Xi’an. (Response)

S1: *他旅行去西安了。* (Response)

T: No. The correct response should be “He went to Xi’an on a trip.” He went to Xi’an on a trip. (Feedback)

T: *不对，应该是他去西安旅行了，去西安旅行。* (Feedback)

S1: He went to Xi’an on a trip. (Response)

S1: *他去西安旅行了。* (Response)

S2: David went to Xi’an on a trip. (Response)

S2: *大卫去西安旅行了。* (Response)

T: Very good! (Feedback) Did Mary go to Xi’an too? (Initiate)

T: *非常好！* (Feedback) *玛丽也去西安了吗？* (Initiate)

S2: No, Mary went to Chengdu on a trip. (Response)

S2: *没有，玛丽去成都旅行了。* (Response)

T: Yes, Mary went to Chengdu on a trip. (Feedback)

T: *对，玛丽去成都旅行了。* (Feedback)

##### Interaction in FonF

The interaction in FonF existed not only among learners but also between teachers and learners. During the interaction between teachers and learners, both sides could initiate new topics through one-on-one interaction. In this interaction, the teacher’s questions were relatively open and had no determined answers (e.g., “what is your first impression of this place?”), which helped learners produce more complex syntactic structures. The teacher directed learners’ attention to the linguistic forms that might cause difficulties in communication, and learners could ask the teacher questions about these linguistic forms. When an error occurred in the learner’s output, the teacher and other learners corrected it using negotiation and feedback. Negotiation included negation of both meaning and form. Feedback included both implicit and explicit feedback. In implicit feedback, recasting was used to help learners identify errors by repeating learners’ sentences or using rising tones. If the learner did not correct the errors, the teacher would correct the answer by repeating the correct sentences. For some difficult syntactic structures, the teacher used the feedback of metalinguistic explanation. Following is an example of one such interaction in FonF:

S: I cleaned the room. It’s very clean. Hmm… So, I clean the room?

S: *我把房间打扫了，很干净。嗯……所以说 我把房间打扫干净？*

T: You clean the room?

T: 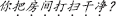


S: (No response)

T: Has the room been cleaned up now?

S: 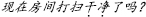


S: It’s clean now.

S: *打扫干净了。*

T: Good. What is the result of “cleaning”?

T: 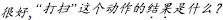


S: I cleaned up the room.

S: *我把房间打扫干净了。*

T: Good. You cleaned up the room.

T: 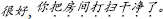


*Note:* The parts highlighted and marked in red are the prosodic focus.

### Instruments

These tests were conducted three times, including a pretest (the day before the first class), immediate posttest (the second day after the last class), and delayed posttest (the seventh day after the last class). To prevent participants from perceiving the purpose of the experiment and reducing the practice effect, participants completed four oral tasks in each test, in which the topics of the tasks were related to the learning content. Two of the four topics appeared repeatedly in the three tests (the target topics), such as *How do you make a travel plan?* and *How do you shop online?* The other six filling topics involved *shopping* (pretest), *career choice* (pretest), *renting* (immediate posttest), *travel way* (immediate posttest), *gift giving* (delayed posttest), and *fitness* (delayed posttest). All topics were coded with numbers. Each participant randomly selected the numbers and completed the chosen tasks in order. The testing task provided the participants with the keywords and syntactic structures of the topic as a reference for oral production.

### Data Collection

The study used the same testing tasks across proficiency levels. Each participant performed a monolog task for 3 min. All participants completed the tests in a quiet classroom. For each testing task, the participants had 3 min to prepare. If they had any questions about the task, they could ask the teacher for help before the test started. There was no time limit for participants to complete the test. The recording equipment was a notebook computer (Asus R417S) and the recording software was PRAAT. The recording sample was 16-bit mono and had a sampling rate of 44,100 Hz.

### Measurement of Dependent Variables

We used analysis of speech units (AS-units) to calculate complexity and accuracy of oral production (Foster et al., 2000, p. 365). Division into AS-units was achieved based on the characteristics of Chinese syntactic structure, referring to the three principles constructed by Chen and Li (2016). According to the first principle, a single sentence with strong independence is regarded as one AS-unit. A single sentence can be a word, phrase, or clause with a significant declined intonation and a long pause at the end. According to the second principle, a complex sentence composed of multiple subclauses is one AS-unit, and a close semantic relationship exists between the subclauses, whether or not the complex sentences use conjunctions. A pause can be observed at the end of a subclause, but it is significantly shorter than the pause at the end of a complex sentence. The declination of the intonation at the end of a subclause is small, and a long pause and significant declined intonation at the end of a complex sentence can be found. According to third principle, a false start, repetition, and self-correction all are calculated as one AS-unit, but these speech components should be deleted when calculating complexity and fluency.

We used two measurements of complexity: syntactic and lexical complexity. Syntactic complexity was measured by the number of subclauses per AS-unit (Yu and Lowie, 2020). Lexical complexity was measured by the Guiraud index (Guiraud, 1954). We calculated the accuracy of the two measurements as the number of lexical errors per AS-unit (lexical errors/AS-units) and the number of syntactic errors per AS-unit (syntactic errors/AS-units; Chen, 2015). We measured fluency by the mean length of run (MLR), which was calculated as the mean number of syllables produced in utterances between pauses of >0.2 s (Towell et al., 1996).

### Transcription and Annotation

We collected 192 recordings of learners’ oral production. Two graduate students with a background in linguistic completed the transcription and annotation of the oral production corpus. The transcription and annotation were completed following two steps: First, the two annotators transcribed as they listened to the recordings. After transcription, they exchanged their transcriptions with each other and checked the consistency. For any inconsistencies, the two annotators negotiated to reach an agreement. Second, the two annotators not only labeled the learners’ oral production for complexity, accuracy, and fluency but also double-checked the annotation results.

## Results

We systematically analyzed the influence of the instruction method on the oral production of Korean native speakers. At the same time, we also analyzed the modulation effects of testing time and L2 proficiency on learning outcomes. On the basis of the normality tests (see Supplementary Appendix 2) and Levene’s test of equality of error variances (see Supplementary Appendix 3), we found that the data of pretest, immediate posttest, and delayed posttest were close to the normal distribution, and the error variance of the dependent variables were equal across groups. Therefore, we conducted a multiway ANOVA. Three independent variables and five dependent variables were involved in this experiment. To verify the existence of multiple variables and their complex relationships, we conducted three-way mixed ANOVA for each dependent variable. The descriptive statistical results of oral production are shown in Table 4.

**TABLE 4:** Descriptive statistical results for oral production.

Oral production		Instruction group	L2 proficiency	Pretest	Immediate posttest	Delayed posttest	*N*
Complexity	Lexical	FonF	L	15.690 (2.592)	17.774 (2.806)	17.964 (2.033)	8
	complexity		H	23.352 (2.361)	24.588 (3.946)	26.961 (3.763)	8
		FonFs	L	15.924 (3.251)	17.268 (3.006)	18.981 (4.030)	8
			H	24.821 (3.176)	25.768 (5.266)	25.363 (6.174)	8
	Syntax	FonF	L	1.729 (0.283)	1.886 (0.166)	1.863 (0.230)	8
	complexity		H	1.928 (0.274)	2.147 (0.230)	2.013 (0.242)	8
		FonFs	L	1.736 (0.248)	1.790 (0.213)	1.773 (0.293)	8
			H	1.962 (0.200)	2.102 (0.106)	1.990 (0.211)	8
Accuracy	Lexical	FonF	L	0.326 (0.118)	0.244 (0.104)	0.160 (0.082)	8
	accuracy		H	0.220 (0.142)	0.218 (0.103)	0.170 (0.087)	8
		FonFs	L	0.294 (0.167)	0.213 (0.066)	0.224 (0.092)	8
			H	0.233 (0.064)	0.157 (0.077)	0.128 (0.062)	8
	Syntactic	FonF	L	0.337 (0.076)	0.267 (0.049)	0.277 (0.052)	8
	accuracy		H	0.278 (0.102)	0.204 (0.079)	0.197 (0.055)	8
		FonFs	L	0.323 (0.179)	0.233 (0.058)	0.251 (0.076)	8
			H	0.289 (0.059)	0.240 (0.095)	0.176 (0.091)	8
Fluency	Mean length of	FonF	L	4.186 (0.445)	5.069 (0.810)	5.149 (0.742)	8
	run		H	6.139 (0.476)	6.693 (0.853)	7.223 (2.014)	8
		FonFs	L	4.007 (0.668)	4.461 (0.501)	4.458 (0.686)	8
			H	6.135 (1.227)	6.215 (0.947)	6.50 (0.889)	8

### The Complexity of Oral Production

The results of three-factor mixed ANOVA (see Table 5) of lexical complexity and syntactic complexity showed that the main effects of the instruction method were not significant with *F*(1,28) = 0.085, *p* = 0.772, $\eta_{\text{p}}^{2}$ = 0.003 and *F*(1,28) = 0.257, *p* = 0.616, and ${\text{η}}_{\text{p}}^{2}$ = 0.009. The main effects of the L2 proficiency, however, were significant with *F*(1,28) = 52.725, *p* = 0.000, ${\text{η}}_{\text{p}}^{2}$ = 0.653 and *F*(1,28) = 10.622, *p* = 0.003, and ${\text{η}}_{\text{p}}^{2}$ = 0.275. The lexical complexity and syntactic complexity of the high-proficiency groups were significantly higher than those of the low-proficiency groups. The main effects of the testing time were significant, with *F*(2,56) = 6.967, *p* = 0.002, ${\text{η}}_{\text{p}}^{2}$ = 0.199 and *F*(2,56) = 7.821, *p* = 0.001, and ${\text{η}}_{\text{p}}^{2}$ = 0.218. Least significant difference (LSD) multiple comparison showed the lexical complexity of the immediate posttest and delayed posttest were significantly higher than the pretest (*p* < 0.05; *p* < 0.01), the lexical complexity of the delayed posttest was significantly higher than the immediate posttest (*p* < 0.05), and the syntactic complexity of the immediate posttest was significantly higher than that of the pretest (*p* < 0.01) and delayed posttest (*p* < 0.05). We did not find a significant difference between the pretest and delayed posttest (*p* > 0.05) of the syntactic complexity. The interactions among the testing time, instruction method, and L2 proficiency were not significant (see Table 5).

**TABLE 5:** Results of three-factor mixed ANOVA for oral production complexity.

Oral production complexity	Source	*df*	*F*	*p*	${\text{η}}_{\text{p}}^{2}$
Lexical complexity	Between subjects				
	Instruction method	1	0.085	0.772	0.003
	L2 proficiency	1	52.725	0.000***	0.653
	Instruction method × L2 proficiency	1	0.004	0.951	0.000
	Error	28			
	Within subjects				
	Testing time	2	6.967	0.002**	0.199
	Instruction method × testing time	2	0.427	0.655	0.015
	L2 proficiency × testing time	2	0.124	0.884	0.004
	Instruction method × L2 proficiency × testing time	2	1.711	0.190	0.058
	Error	56			
Syntactic complexity	Between subjects				
	Instruction method	1	0.257	0.616	0.009
	L2 proficiency	1	10.622	0.003**	0.275
	Instruction method × L2 proficiency	1	0.119	0.732	0.004
	Error	28			
	Within subjects				
	Testing time	2	7.821	0.001**	0.218
	Instruction method × testing time	2	0.930	0.400	0.032
	L2 proficiency × testing time	2	1.081	0.346	0.037
	Instruction method × L2 proficiency × testing time	2	0.037	0.964	0.001
	Error	56			

** p < 0.01; *** p < 0.001.

### The Accuracy of Oral Production

The results of three-way mixed ANOVA of lexical accuracy and syntactic accuracy showed that the main effects of the instruction method were not significant with *F*(1,28) = 0.260, *p* = 0.614, ${\text{η}}_{\text{p}}^{2}$ = 0.009 and *F*(1,28) = 0.110, *p* = 0.742, and ${\text{η}}_{\text{p}}^{2}$ = 0.004. The main effects of the L2 proficiency were marginally significant with *F*(1,28) = 3.568, *p* = 0.069, and ${\text{η}}_{\text{p}}^{2}$ = 0.113 on lexical accuracy and were significant with *F*(1,28) = 4.261, *p* = 0.048, and ${\text{η}}_{\text{p}}^{2}$ = 0.132 on syntactic accuracy. The lexical accuracy and the syntactic accuracy of the high-proficiency groups were better than that of the low-proficiency groups. The main effects of the testing time were significant with *F*(2,56) = 15.266, *p* < 0.001, ${\text{η}}_{\text{p}}^{2}$ = 0.353 and *F*(2,56) = 14.595, *p* < 0.001, and ${\text{η}}_{\text{p}}^{2}$ = 0.343. LSD multiple comparison showed that the lexical accuracy of the immediate posttest and delayed posttest was significantly better than the pretest (*p* < 0.01, *p* < 0.001), and the lexical accuracy of the delayed posttest was significantly better than the immediate posttest (*p* < 0.01), and the syntactic accuracy of the immediate posttest and delayed posttest was significantly better than that of the pretest (*p* < 0.001, *p* < 0.01). We did not find a significant difference between the immediate posttest and delayed posttest (*p* > 0.05) of the syntactic accuracy. The interactions among the testing time, instruction method, and L2 proficiency were not significant (see Table 6).

**TABLE 6:** Results of three-factor mixed ANOVA for oral production accuracy.

Oral production accuracy	Source	*df*	*F*	*p*	${\text{η}}_{\text{p}}^{2}$
Lexical accuracy	Between subjects				
	Instruction method	1	0.260	0.614	0.009
	L2 proficiency	1	3.568	0.069*	0.113
	Instruction method × L2 proficiency	1	0.257	0.616	0.009
	Error	28			
	Within subjects				
	Testing time	2	15.266	0.000***	0.353
	Instruction method × testing time	2	1.311	0.278	0.045
	L2 proficiency × testing time	2	0.095	0.410	0.031
	Instruction method × L2 proficiency × testing time	2	2.236	0.116	0.074
	Error	56			
Syntactic accuracy	Between subjects				
	Instruction method	1	0.110	0.742	0.004
	L2 proficiency	1	4.261	0.048*	0.132
	Instruction method × L2 proficiency	1	0.462	0.502	0.016
	Error	28			
	Within subjects				
	Testing time	2	14.595	0.000***	0.343
	Instruction method × testing time	2	0.331	0.720	0.012
	L2 proficiency × testing time	2	1.188	0.312	0.041
	Instruction method × L2 proficiency × testing time	2	0.526	0.594	0.018
	Error	56			

* p < 0.1; *** p < 0.001.

### The Fluency of Oral Production

The results of three-way mixed ANOVA of fluency showed that the main effect of the instruction method was marginally significant, with *F*(1,28) = 3.666, *p* = 0.066, and ${\text{η}}_{\text{p}}^{2}$ = 0.116, and the mean length of run of the FonF group was longer than that of the FonFs group. The main effect of the L2 proficiency was significant, with *F*(1,28) = 46.208, *p* = 0.000, and ${\text{η}}_{\text{p}}^{2}$ = 0.623. The mean length of run of the high-proficiency groups was significantly longer than that of the low-proficiency groups. The main effect of the testing time was significant with *F*(2,56) = 7.269, *p* = 0.002, and ${\text{η}}_{\text{p}}^{2}$ = 0.206. LSD multiple comparison showed that the mean lengths of run of the immediate posttest and delayed posttest were significantly longer than the mean lengths of run of the pretest (*p* < 0.001; *p* < 0.01). We did not find, however, any significant difference between the immediate posttest and delayed posttest (*p* > 0.05). The interaction between the testing time and instruction method was marginally significant, with *F*(2,56) = 3.118, *p* = 0.05, and ${\text{η}}_{\text{p}}^{2}$ = 0.100. The other interactions were not significant (see Table 7).

**TABLE 7:** Results of three-factor mixed ANOVA for oral production fluency.

Oral production fluency	Source	*df*	*F*	*p*	${\text{η}}_{\text{p}}^{2}$
Mean length of run	Between subjects				
	Instruction method	1	3.666	0.066*	0.116
	L2 proficiency	1	46.208	0.000***	0.623
	Instruction method × L2 proficiency	1	0.012	0.915	0.000
	Error	28			
	Within subjects				
	Testing time	2	7.269	0.002**	0.206
	Instruction method × testing time	2	3.118	0.05*	0.100
	L2 proficiency × testing time	2	0.551	0.580	0.019
	Instruction method × L2 proficiency × testing time	2	0.594	0.556	0.021
	Error	56			

* p < 0.1; ** p < 0.01; *** p < 0.001.

**FIGURE 1:**
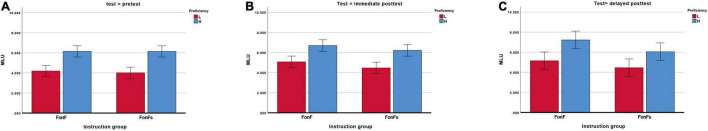
Effects of different instruction methods on fluency (MLU). **(A)** The MLU of the pretest. **(B)** The MLU of the immediate posttest. **(C)** The MLU of the delayed posttest.

Because we observed a marginal significant interaction between the instruction method and the testing time, we carried out simple effect tests (see Figure 1). We found a significant effect on the mean length of run of the FonF group, with *F*(2,27) = 8.726, *p* = 0.001, and ${\text{η}}_{\text{p}}^{2}$ = 0.393. The multiple comparisons showed that the mean length of run of the immediate posttest and delayed posttest was significantly longer than that of the pretest (*p* < 0.001; *p* < 0.01). However, the difference between the immediate posttest and delayed posttest was not significant (*p* > 0.05). The effect of the testing time on mean length of run in the FonFs group was not significant, with *F*(2,27) = 1.460, *p* = 0.250, and ${\text{η}}_{\text{p}}^{2}$ = 0.098. The effects of the instruction method on the mean length of run for the immediate posttest and delayed posttest were both significant. The mean length of run of the FonF group was marginally or significantly higher than that of the FonFs group, with *F*(1,28) = 3.734, *p* = 0.06, and ${\text{η}}_{\text{p}}^{2}$ = 0.118 and *F*(1,28) = 4.737, *p* = 0.038, and ${\text{η}}_{\text{p}}^{2}$ = 0.145.

### Analysis of Oral Production of Different L2 Proficiency

We carried out multiple one-way ANOVA to further analyze the effects of three testing times on different proficiency learners with different instruction methods. The results showed that the testing time significantly affected the lexical accuracy, with *F*(2,24) = 4.363, *p* < 0.05, and ${\text{η}}_{\text{p}}^{2}$ = 0.294 and mean length of run with *F*(2,24) = 4.872, *p* < 0.05, and ${\text{η}}_{\text{p}}^{2}$ = 0.317 of low-proficiency learners in the FonF group. The testing time also significantly affected the lexical accuracy with *F*(2,24) = 5.779, *p* < 0.05, and ${\text{η}}_{\text{p}}^{2}$ = 0.355 and syntactic accuracy with *F*(2,24) = 3.744, *p* < 0.05, and ${\text{η}}_{\text{p}}^{2}$ = 0.263 of high-proficiency learners in the FonFs group. In the FonF group, for low-proficiency learners, the lexical accuracy of delayed posttest was significantly higher than that of pretest (*p* < 0.05), and the mean length of run of immediate posttest and delayed posttest was significantly higher or longer than that of pretest (*p* < 0.05). In the FonFs group, for high-proficiency learners, the lexical accuracy and syntactic accuracy of delayed posttest were significantly higher than that of pretest (*p* < 0.01; *p* < 0.05).

## Discussion

After Korean native speakers took five classes, their complexity, accuracy, and fluency of L2 Chinese oral production was significantly improved in both instruction groups. The effects of the instruction methods on these three dimensions of oral production differed, however. Additionally, in different tests, the learning effects of the different instruction methods also were different and were modulated by L2 proficiency. Therefore, we examined the influence of different instruction methods on the CAF of L2 Chinese oral production in different tests, as well as the modulation effects of L2 proficiency on learning outcomes.

### The Influence of FonFs and FonF on L2 Chinese Oral Production

The influence of instruction methods on the three dimensions of L2 Chinese oral production differed. FonFs and FonF had no significant differences in complexity and accuracy but yielded a significant difference in fluency. Fluency in the immediate posttest and delayed posttest for the FonF group was marginally or significantly higher than that in the FonFs group. This observation demonstrated that, compared with the FonFs group, FonF can cultivate learners’ selective attention to the linguistic meaning (Doughty, 2001) with a good retention effect, and thus can improve fluency. Interestingly, we did not find a significant difference in complexity and accuracy between the FonF group and the FonFs group, which meant that the improvement of fluency may not be at the cost of accuracy and complexity, and FonF enabled learners, whether intentionally or unintentionally, to notice the linguistic forms.

NH (Schmidt, 1990, 1994) emphasized the importance of paying conscious attention to linguistic forms in language acquisition. IH (Long, 1996) advocated to create communicative needs through interaction in language instruction, thus to make learners aware of the defects of their oral production when they encountered communicative barriers (Swain, 1998; Fuente and Maria, 2006; Spada and Lightbown, 2008). Furthermore, IPH suggested that if teaching method enables learners to construct appropriate connection between the linguistic forms and the linguistic meaning, then it might improve the processing effect of output (VanPatten et al., 2015). In this study, we found that FonF allowed the learners to notice the linguistic forms intentionally or incidentally when they noticed linguistic meaning, therefore to construct the connection between the linguistic form and the linguistic meaning. For example, in the pre-task stage of FonF, learners obtained meaning negotiation by completing the information gap task to focus on the meaning of language. In the task-processing stage, learners not only obtained rich comprehensible input by meaning negotiation but also strengthened attention to the linguistic forms by form negotiation. In the language-focused stage, learners strengthened attention to the linguistic forms with enhanced learning materials. FonFs, however, focused on teachers’ instruction and controlled practice and did not create real communicative needs at different teaching stages. The learners lacked the opportunity of meaning negotiation, resulting in no significant improvement in fluency. Regarding feedback, FonFs adopted only direct correction, whereas FonF adopted feedback that was dominated by recasting and that was supplemented by metalinguistic interpretation. The strategy of integrating explicit and implicit feedbacks helped learner notice the complexity and accuracy of the linguistic forms. FonF was found to enable learners to obtain sufficient meaning negotiation and form negotiation, which is helpful for constructing the connection between the linguistic meaning and form. This observation is consistent with previous studies that a teaching approach with rich interaction and meaning-focused activities is beneficial to fluency (Sato and Lyster, 2012; Arslanyilmaz, 2013; Pena and Pladevall-Ballester, 2020; Hyun, 2021).

### The Interaction Between Instruction Method and Testing Time

The interaction between the instruction method and the testing time had a significant effect on fluency only. The simple effect test showed that a significant effect was observed on fluency in the FonF group, whereas no significant effect was found in the FonFs group. Fluency in the immediate posttest and delayed posttest for the FonF group was significantly higher than that of the pretest, although no significant difference was observed between the two posttests. We did not observe any significant difference in fluency of the FonFs group among the three tests. These results indicated that the fluency of the FonF group had been significantly improved, resulting in a greater learning effect and good retention effect. In contrast, the fluency of the FonFs group was not significantly improved. Previous studies have found that FFI can improve L2 fluency, accuracy, and the ability to use the more complex linguistic forms (e.g., Spada and Lightbown, 1993; Doughty and Varela, 1998; Lyster, 2004), but the FonF was more effective in improving fluency (Spada and Lightbown, 2008). In this study, we found that learners could notice the linguistic meaning in the FonF group, which promoted the significant development of the fluency. Unlike FonF, FonFs made learners pay more attention to the linguistic forms rather than to linguistic meaning. According to NH (Schmidt, 2001), the probability of internalization of a language feature with insufficient attention would be reduced. Here, we found that the significant improvement of fluency in FonF group in two post tests, which indicated the learners did notice the linguistic meaning. However, unlike FonF, there was no significant improvement in fluency in FonFs group, which suggested that the learners paid more attention to the linguistic forms and resulted in a lower automaticity of oral production.

The interaction between the instruction method and the testing time had no significant impact on complexity and accuracy, whereas the tests had a significant impact on the complexity and the accuracy of both groups. In terms of lexical complexity and accuracy, performance in the immediate posttest and delayed posttest was significantly better than in the pretest, and the performance of the delayed posttest was significantly better than the immediate posttest, which indicated that the learning effects were well maintained. For syntactic complexity and syntactic accuracy, performance in the immediate posttest was significantly better than the pretest, the delayed posttest was not significantly different from the pretest, which indicated that the learning effects remained poor. The differences between the immediate posttest and the delayed posttest in syntactic accuracy were not significant, however, which indicated that the learning effects were maintained well. In summary, no matter what kind of instruction conditions were used for the learners, the learning effects for lexical forms were maintained better than the syntactic forms, and the learning effects for syntactic complexity were the worst. The explanation for this is that syntactic abilities, such as sentence structure organization, cohesive construction, and semantic integration were more difficult to acquire because of their weak explicitness (Ahmadian and Tavakoli, 2011; Ellis, 2012; Chen and Li, 2016; Kang et al., 2019), and more cognitive efforts were needed to increase the number of AS-unit clauses. Therefore, it is necessary to explore the relationship between learners’ attention to syntactic complexity and oral production. The exploration will help to design divergent teaching approaches, which enable learners to better establish the mapping relationships between syntactic complexity and its meaning.

### The Modulation Effects of L2 Proficiency

In this study, FonF was found to be more helpful to promote lexical accuracy and fluency of low-proficiency learners, whereas FonFs was more beneficial to improve the accuracy of high-proficiency learners. In FonF, lexical accuracy had delayed learning effect, and the mean length of run had immediate and delayed learning effect for low-proficiency learners. We also found, however, delayed learning effects on lexical accuracy and syntactic accuracy for high-proficiency learners in FonFs. These results indicated that L2 proficiency was able to modulate the relationship between instruction methods and outcomes to a certain extent (Spada and Lightbown, 2008; Kang et al., 2019).

Compared with FonF, high-proficiency learners paid more attention to the linguistic forms in FonFs. FonFs highlights the focus on the linguistic forms, which leads high-proficiency learners to encounter fewer difficulties in decoding and encoding of linguistic meaning by paying more attention to the linguistic forms (see VanPatten, 1990). Additionally, the emphasis for the linguistic forms in FonFs is conducive to the development of the accuracy of representing explicit knowledge. Compared with high-proficiency learners, low-proficiency learners need more external supports for their lack of L2 knowledge and skill (Kang et al., 2019). In this study, we found that FonF paid more attention to the linguistic meaning compared with FonFs. Therefore, it was helpful to pay more attention to the linguistic meaning for low-proficiency learners who have greater difficulties in decoding and encoding linguistic meaning, which was conducive to the development of fluency that reflected implicit knowledge. In addition, FonF paid attention to the linguistic forms while also noticing meaning, which helped low-proficiency learners notice the linguistic forms to a certain extent, such as lexical accuracy. It is noteworthy that the existing traditional L2 Chinese instruction pays more attention to the linguistic forms in the elementary level, while the instruction in the advanced level pays more attention to the expression of the linguistic meaning (Gong et al., 2020b,^2021^). However, low-proficiency learners should also accept instructions that pay attention to both linguistic meaning and linguistic forms. And, the training of the linguistic forms can never be ignored and should be carried out iteratively for high-proficiency learners.

## Conclusion

In this study, we investigated the effects of different instruction methods of FFI, including FonF and FonFs, on the complexity, accuracy, and fluency of oral production of native Korean speakers’ L2 Chinese. We found that FonF paid attention to both linguistic meaning and linguistic form, which achieved significant learning effects on fluency while also kept accuracy and complexity. FonFs, however, mainly focused on the linguistic forms, which were not conducive to the improvement of fluency because fluency did not obtain significant immediate and delayed learning effects. We also found that the outcome measures had modulation effects on learning effects. The learning effects of the lexical forms were better than those of syntactic forms, whether in FonF or FonFs. Last, we found that L2 proficiency had modulation effects on learning effects. The oral fluency of low-proficiency learners benefited the most from FonF, whereas the oral accuracy of high proficiency learners benefited the most from FonFs.

The findings of this study have some practical implications for the instruction of L2 spoken Chinese. For instance, teachers should adopt appropriate approaches to help learners construct the connection between linguistic forms and linguistic meaning to improve oral production. In FonF, teachers can create communicative needs by using multiple negotiation and feedback strategies. Thus, learners can realize the gap between their own language and native speakers of the target language when they encounter communicative obstacles. Doing so will promote attention to the linguistic forms. Our findings are a reminder that teachers should be aware that learners gain different benefits from different instruction methods because of different L2 proficiency. Low-proficiency learners better accept instruction methods that pay attention to both linguistic form and linguistic meaning, which are beneficial for learners to build connections between linguistic form and linguistic meaning during the elementary stage of L2 learning. For high-proficiency learners, instruction should be carried out iteratively. It is not sufficient to pay attention only to the language meaning, and the linguistic forms also should be given continuous attention. In this way, learners can carry out in-depth learning from their personal learning experiences with the linguistic forms and can further internalize the connection between linguistic meaning and linguistic form. In addition, the outcome measures can modulate these learning effects, which remind us that the learning effects of different instruction methods are different with different types of knowledge measured. Therefore, global assessments should be used to explore the impact of different types of FFI on these different oral dimensions.

Note that this study has some limitations. For example, the sample size of participants was relatively small. Each of the four experimental groups had only eight participants. The implementation time for each teaching experiment was only 2 weeks, which should be extended in future studies. Moreover, there was no qualitative analysis of learners’ attitudes toward different instruction methods. Therefore, in the future, we will recruit more participants from different L1 backgrounds to implement teaching methods for a longer timeline, and will use reflective journals and semi-structured interviews to investigate L2 Chinese learners’ attitudes toward different instruction conditions. We believe that a more comprehensive study could provide a multidimensional perspective for the scientific implementation of these instruction methods.

## Data Availability Statement

The original contributions presented in the study are included in the article/Supplementary Material, further inquiries can be directed to the corresponding author.

## Ethics Statement

The studies involving human participants were reviewed and approved by Institutional Reviewing Board (IRB) of Beijing Language and Culture University. The patients/participants provided their written informed consent to participate in this study.

## Author Contributions

MC and WL conceived and designed the study. MC drafted and revised the manuscript and got it ready for submission. WL collected the data. Both authors contributed to the article and approved the submitted version.

## Conflict of Interest

The authors declare that the research was conducted in the absence of any commercial or financial relationships that could be construed as a potential conflict of interest.

## Publisher’s Note

All claims expressed in this article are solely those of the authors and do not necessarily represent those of their affiliated organizations, or those of the publisher, the editors and the reviewers. Any product that may be evaluated in this article, or claim that may be made by its manufacturer, is not guaranteed or endorsed by the publisher.
