# Supplementary material for: The Influence of Form-Focused Instruction on the L2 Chinese Oral Production of Korean Native Speakers
Source: Front Psychol. 2022 Apr 11;13:790424. doi: 10.3389/fpsyg.2022.790424 (PMC9035550; doi:10.3389/fpsyg.2022.790424)
Supplement: Supplementary file 1 [file Data_Sheet_1.docx]

**APPENDICES**

**Appendix 1 | The Example of Learning Materials**

- **Learning Materials for Travel Planning of Low-proficiency Learners**

下周就是国庆节了，我和我的朋友计划去南方玩几天。我们想去四川成都、浙江杭州还有云南大理。因为我们对这些地方不太熟悉，所以我们打算请一位导游带着我们旅行。

我们10月1日从北京首都国际机场出发，先去杭州看西湖美景、吃小吃。然后再从杭州坐高铁去成都和大理。听说成都的火锅和大理的手工艺品都很有名，我们一定要尝尝地道的成都火锅，还要在大理买一些小礼物带回来。

我们已经在网上买好了景区的门票，因为假期游客很多，这样就不用排队买票了。我们都希望这是一次愉快的旅行。

- **Learning Materials for Travel Planning of High-proficiency Learners**

十一黄金周要到了，我和我的朋友计划游览四川成都、浙江杭州以及云南大理。因为对当地不太熟悉，所以我们打算跟团游，而不是自助游，住宿由导游为我们安排。

我们10月1日从北京首都国际机场出发，先去杭州游览西湖美景、品尝小吃。之后会坐高铁去成都和大理。听说成都的火锅和大理的手工艺品很有特色，所以除了游览以外，我们还要尝尝地道的成都火锅，再买一些纪念品带给亲朋好友。

现在是旅行旺季，景区一般很拥挤，所以我们在网上预订了景区的门票。我们都盼着这一天快点到来。

Note: the parts with straight underlines are new words, and the parts with wavy underlines are gramma points.

**Appendix 2 | The Tests of Normality**

| Oral production | | Testing time | Statistic | Shapiro-wilk | |
| --- | --- | --- | --- | --- | --- |
|  |  |  |  | N | Sig. |
| Complexity | Lexical  complexity | Pretest | .957 | 32 | .230 |
|  |  | Immediate posttest | .954 | 32 | .187 |
|  |  | Delayed posttest | .957 | 32 | .234 |
|  | Syntax  complexity | Pretest | .972 | 32 | .563 |
|  |  | Immediate posttest | .958 | 32 | .248 |
|  |  | Delayed posttest | .983 | 32 | .875 |
| Accuracy | Lexical  accuracy | Pretest | .973 | 32 | .597 |
|  |  | Immediate posttest | .942 | 32 | .085 |
|  |  | Delayed posttest | .941 | 32 | .080 |
|  | Syntactic  accuracy | Pretest | .979 | 32 | .757 |
|  |  | Immediate posttest | .977 | 32 | .723 |
|  |  | Delayed posttest | .960 | 32 | .279 |
| Fluency | Mean length of run | Pretest | .938 | 32 | .066 |
|  |  | Immediate posttest | .941 | 32 | .080 |
|  |  | Delayed posttest | .975 | 32 | .642 |

**Appendix 3 | Levene's Test of Equality of Error Variances (Based on Mean)**

| Oral production | | Testing time | Levene Statistic | df1 | df2 | Sig. |
| --- | --- | --- | --- | --- | --- | --- |
| Complexity | Lexical  complexity | Pretest | 0.661 | 3 | 28 | 0.583 |
|  |  | Immediate posttest | 2.625 | 3 | 28 | 0.070 |
|  |  | Delayed posttest | 2.446 | 3 | 28 | 0.085 |
|  | Syntax  complexity | Pretest | 0.142 | 3 | 28 | 0.934 |
|  |  | Immediate posttest | 1.749 | 3 | 28 | 0.180 |
|  |  | Delayed posttest | 0.617 | 3 | 28 | 0.610 |
| Accuracy | Lexical  accuracy | Pretest | 1.127 | 3 | 28 | 0.355 |
|  |  | Immediate posttest | 0.958 | 3 | 28 | 0.426 |
|  |  | Delayed posttest | 0.780 | 3 | 28 | 0.515 |
|  | Syntactic  accuracy | Pretest | 2.510 | 3 | 28 | 0.079 |
|  |  | Immediate posttest | 2.500 | 3 | 28 | 0.080 |
|  |  | Delayed posttest | 1.092 | 3 | 28 | 0.369 |
| Fluency | Mean length of run | Pretest | 2.523 | 3 | 28 | 0.078 |
|  |  | Immediate posttest | 1.266 | 3 | 28 | 0.305 |
|  |  | Delayed posttest | 2.823 | 3 | 28 | 0.057 |
